# CyberKnife Xsight versus fiducial-based target-tracking: a novel 3D dosimetric comparison in a dynamic phantom

**DOI:** 10.1186/s13014-022-02123-1

**Published:** 2022-09-08

**Authors:** Thomas J. Klein, Suki Gill, Martin A. Ebert, Garry Grogan, Warwick Smith, Zaid Alkhatib, John Geraghty, Alison J. D. Scott, Alan Brown, Pejman Rowshanfarzad

**Affiliations:** 1 School of Physics, Mathematics and Computing, The University of Western Australia, 35 Stirling Highway, Mailbag M013, Crawley, WA 6009 Australia; 2 Department of Radiation Oncology, Sir Charles Gairdner Hospital, Nedlands, WA Australia; 3 Radiotherapy Physics Department, Churchill Hospital, Old Road, Headington, Oxford UK

**Keywords:** CyberKnife, Tumor-tracking, Xsight, 3D Dosimetry

## Abstract

**Background:**

The CyberKnife Xsight lung-tracking system (XLTS) provides an alternative to fiducial-based target-tracking systems (FTTS) for non-small-cell lung cancer (NSCLC) patients without invasive fiducial insertion procedures. This study provides a method for 3D independent dosimetric verification of the accuracy of the FTTS compared to the XLTS without relying on log-files generated by the CyberKnife system.

**Methods:**

A respiratory motion trace was taken from a 4D-CT of a real lung cancer patient and applied to a modified QUASAR™ respiratory motion phantom. A novel approach to 3D dosimetry was developed using Gafchromic EBT3 film, allowing the 3D dose distribution delivered to the moving phantom to be reconstructed. Treatments were planned using the recommended margins for one and three fiducial markers and XLTS 2-view, 1-view and 0-view target-tracking modalities. The dose delivery accuracy was analysed by comparing the reconstructed dose distributions to the planned dose distributions using gamma index analysis.

**Results:**

For the 3%/2 mm gamma criterion, gamma passing rates up to 99.37% were observed for the static deliveries. The 3-fiducial and 1-fiducial-based deliveries exhibited passing rates of 93.74% and 97.82%, respectively, in the absence of target rotation. When target rotation was considered, the passing rate for 1-fiducial tracking degraded to 91.24%. The passing rates observed for XLTS 2-view, 1-view and 0-view target-tracking were 92.78%, 96.22% and 76.08%, respectively.

**Conclusions:**

Except for the XLTS 0-view, the dosimetric accuracy of the XLTS was comparable to the FTTS under equivalent treatment conditions. This study gives us further confidence in the CyberKnife XLTS and FTTS systems.

## Background

Stereotactic ablative radiation therapy (SABR) has been shown to provide similar survival rates to surgery for stage 1 non-small-cell lung-cancer (NSCLC) patients based on the pooled analysis of the STARS and ROSEL trials, as well as superior local control compared to standard radiotherapy in the CHISEL study [1, 2]. The CyberKnife robotic radiosurgery system is a frameless SABR system equipped with two real-time respiratory tracking and compensation systems, a fiducial-based target-tracking system (FTTS), as well as the Xsight lung tracking system (XLTS). When the FTTS is employed, one or more fiducial markers will be inserted percutaneously, which carries an associated risk of pneumothorax between 13 and 45%, as well as a risk of haemorrhage, with the risk increasing with the number of fiducial insertions [3, 4]. The use of at least three fiducial markers is considered to be ideal for target-tracking, as this allows for correction of both tumour rotation and translation, minimizing targeting errors and allowing for intra-fractional corrections to be made with 6 degrees of freedom [5]. Conversely, the XLTS involves the direct imaging of the tumour for target-tracking, utilizing pattern-similarity intensity matching algorithms to obtain the positional information of the tumour through comparison of orthogonal X-ray images acquired at the time of treatment with digitally reconstructed radiographs obtained during treatment planning [6]. As a result, the XLTS utilizes soft tissue tracking and does not require any invasive fiducial marker insertion procedures [7].

A number of studies have investigated the tracking accuracy associated with either the XLTS or the FTTS, either via retrospective analysis based on clinical data, or using a phantom model [8–13]. A majority of these studies utilized the log-files generated internally by the CyberKnife system during treatment delivery in order to conduct their analysis, and consequently, these studies are unable to provide an independent verification of the accuracy of the XLTS when compared to the FTTS. Nioutsikou et al. [11] verified the dosimetric accuracy of the Synchrony system for the case of the FTTS, but no comparison between the accuracy of the FTTS and the XLTS was made [11]. Jung et al. [14] investigated the dosimetric accuracy of the CyberKnife XLTS when compared to the FTTS [14]. Despite the excellent results observed in this study, fiducial-based target-tracking was conducted with a single fiducial marker and a spine setup was not possible with the phantom used for this study. As a result, spine alignment could not be completed for both the XLTS and fiducial-based deliveries and a large number of assumptions would have had to be made about the phantom setup and alignment which would not apply in a real-life clinical environment.

The aim of this study is to present a dosimetric methodology to evaluate the accuracy of plan deliveries when using XLTS tracking through comparisons with the fiducial tracking system results.

## Materials and methods

### Phantom design and modifications

**Fig. 1:**
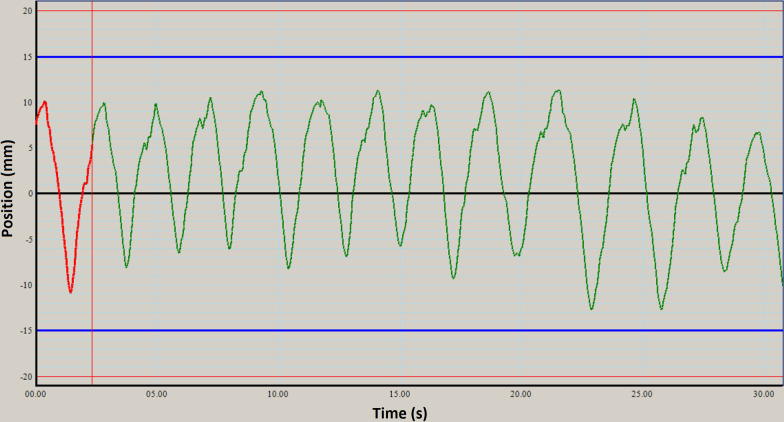
The breathing trace used in this study. All treatment deliveries were completed using the same breathing trace

A QUASAR™ respiratory motion phantom (Modus Medical Devices, London, ON) was programmed to simulate real-patient respiratory breathing pattern. A single breathing pattern exhibiting irregularities in both phase and amplitude was used for all treatment deliveries (Fig. 1).

A thoracic spine segment was 3D printed from poly-lactic acid based on patient CT data available via an open access medical image repository using a Creality-10-S5 printer (Creality3D®, Sydney, NSW). This spine segment was printed hollow and in two halves and was filled with a bone-equivalent material which was prepared by adding a plaster of Paris (CaSO_4_) suspension to a mixture of Eurosil 10 Orange to mimic the CT number of a typical human spine. The spine was then fixed rigidly posterior to the lung insert using a support structure designed to minimize backscatter, allowing the spine scan to serve as a rigid reference to reduce the inter-fractional errors associated with treatment delivery. Using another batch of the Eurosil 10 Orange mixture, a set of ribs and a sternum were also moulded and affixed to the QUASAR phantom utilising a similar determination of the CT number as for the spine. This modification was made to the phantom in order to replicate the attenuation caused by the bony ribcage structure during the acquisition of X-ray images on the CyberKnife system, as this is an effect which could affect the segmentation accuracy when soft-tissue tracking is employed by the XLTS. Lastly, a custom lung insert compatible with the QUASAR respiratory motion phantom was made, consisting of alternating layers of a near-air-equivalent corrugated plastic material. This insert allowed for dose measurements to be taken across 15 layers of Gafchromic EBT3 film spaced 3.28 mm apart, enabling the reconstruction of the 3D dose distribution within the lung volume. An elliptical target with an average diameter of approximately 2.75 cm and a CT number of 100 was also fixed within the lung insert to facilitate target tracking. The gold fiducial markers used in this study were 5 mm long and 0.8 mm thick, and were spaced more than 18 mm apart in all directions based on the departmental clinical protocol.

### Treatment planning

All treatment plans were prepared using the Accuray Precision v1.1 treatment planning system (TPS) (Accuray, Sunnyvale, CA) and delivered using the CyberKnife M6 system (Accuray, Sunnyvale, CA) with a variable aperture iris collimator. A total of 9 treatment plans were prepared for delivery as summarised in Table 1, including a number of static deliveries and treatments delivered with both 1 and 3 fiducial markers for the FTTS. For the XLTS, separate plans were created using different CTV to PTV expansions for a 2-view, 1-view and 0-view treatment setup, which refer to whether the target can be adequately visualised by both, one, or neither of the orthogonal X-ray imagers, depending on possible obstruction of the target by bony anatomy. If neither of the X-ray imagers can adequately visualise the soft tissue target, then a larger treatment margin is employed for a 0-view delivery, with no compensation for tumour movement. Treatment plans were also prepared for delivery to a static target, using both the FTTS and XLTS for setup and alignment, to serve as a baseline to which the other deliveries could be compared. To assess the effect of target rotation on the tracking accuracy, two different phantom setups were employed. In the first setup, the lung insert was not able to rotate about a cranio-caudal axis, and consequently, target-motion was restricted to the superior-inferior direction, while in the second setup, the lung insert was allowed to rotate, adding a component of anterior posterior and left–right target motion. The phantom rotation was a function of the breathing trace and was 30 degrees maximum. For all treatment deliveries, a single fraction dose of 675 cGy was prescribed to the PTV and the dose prescription point was updated to ensure a minimum of 95% PTV coverage was achieved. For all fiducial-based treatment deliveries, a 5 mm isotropic CTV-PTV margin was employed. For the XLTS, the CTV-PTV margins depended on whether the tumour was visible in both orthogonal cameras (2-view), one camera (1-view) or neither camera (0-view). For the 2-view, 5 mm isotropic CTV-PTV margins were employed. With the 1-view, an ITV was generated to account for additional uncertainties in target location using an inhale-exhale planning CT protocol, which was expanded by 7 mm in the untracked directions, and 5 mm in the tracked directions. For the XLTS 0-view, 7 mm margins were applied in all directions to the ITV.

**Table 1:** Summary of treatment plans prepared for each of the different target tracking modalities. The treatment modality, whether Synchrony tracking was employed, and the phantom setup used are indicated. The distance between the imaging isocenter and the target centroid was the same for all experiments

Treatment modality	Synchrony tracking	Phantom setup
3-Fiducial static (FTTS)	No	No target rotation
3-Fiducial synchrony (FTTS)	Yes	No target rotation
1-Fiducial synchrony (FTTS)	Yes	No target rotation
3-Fiducial static (FTTS)	No	Target rotation allowed
1-Fiducial synchrony (FTTS)	Yes	Target rotation allowed
XLTS 2-view static	No	Target rotation allowed
XLTS 2-view	Yes	Target rotation allowed
XLTS 1-view	Yes	Target rotation allowed
XLTS 0-view	No	Target rotation allowed

Planning CT scans were acquired using a Toshiba Aquilion 16-slice Large Bore CT scanner (Toshiba Medical Systems, Otawara, Tochigi, Japan), using the inhale-exhale CyberKnife lung protocol. The exhale CT scan was designated as the primary reference. Calculation settings were the same as clinical practice and all plans were calculated at full CT resolution, representing a voxel size of 1.07 mm × 1.00 mm × 1.07 mm. Dose calculations were performed using a Monte-Carlo algorithm with an uncertainty set to 1%.

### Dosimetric measurements

#### Film measurements and analyses

All dosimetric measurements were carried out using Gafchromic EBT3 film (Ashland Advanced Materials, Bridgewater NJ) from the same batch and were handled according to the recommendations of the AAPM Task Group 55 report [15]. Films were calibrated in the largest field (using the 60 mm diameter collimator). All film measurements were repeated twice and scanned on two different scanners. The calculated uncertainty in film processing and calibration was 0.6%.

Films were digitised 48 h after irradiation with a resolution of 75 dpi using an Epson Expression 10000XL scanner (Seiko Epson Corporation, Nagano) before the images were processed using an in-house developed MATLAB (R2018b, Mathworks, Natick MA) algorithm for film analyses.

**Fig. 2:**
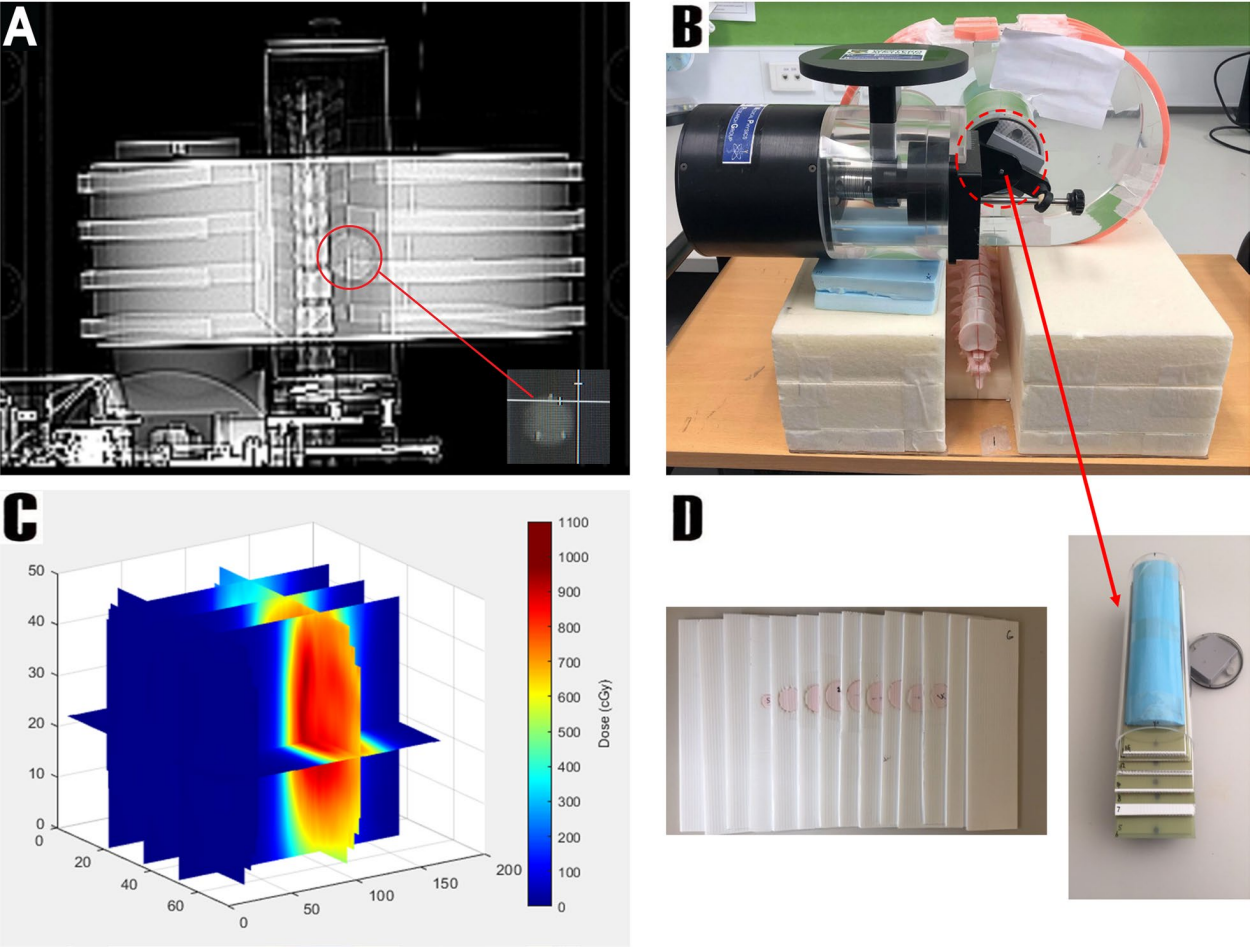
**A** CT Scan of the QUASAR™ respiratory motion phantom with sternum removed to visualise the target. The target with fiducial markers is also shown more clearly; **B** Image of the phantom setup as used for treatment delivery; **C** An example of the reconstructed 3D dose distribution delivered to the lung insert during treatment delivery; **D** The lung insert used for treatment delivery, illustrating the alternating layers of air-equivalent material with the target embedded and Gafchromic EBT3 film for dose measurement

This algorithm utilized the signal measured across the 15 parallel film layers and their known geometry within the lung insert to reconstruct the 3D delivered dose distribution. To ensure accurate alignment of the films, two circles were irradiated perpendicularly on the film layers using a 5 mm stereotactic collimator immediately prior to each delivery. These circles are visible in Fig. 2D. As the film planes were stacked parallel within the lung insert, the centre of each circle had to be in the same position on each film plane and could hence serve as a reference.

The relative alignment of the film layers by the algorithm was first achieved by precisely finding the centre of each circle in each film layer using the circular Hough transform and then determining the equation of a line connecting the centres of the two irradiated circles for each film layer. The algorithm recovered the alignment of films by rotating them to minimise the angle of this line with respect to the central film layer. Once the films were aligned, for each pixel position, the signal along the vertical line bisecting each other film layer orthogonally was interpolated by fitting to the values along that line with a polynomial function of order 9 for most points. Due to the circular nature of the lung insert, film layers within the insert could not be cut to the same size. Consequently, prior to interpolation of the film response, it was necessary to pad the edges of each film image with zero values to ensure the matrices used for interpolation were the same size. These zero values were not included in the interpolation but served as spatial placeholders in the interpolation matrix, ensuring the interpolated film signal was spatially accurate. It should be noted that fitting a polynomial of order 9 to a set of data, required a minimum of 10 datapoints. Therefore, towards the periphery of the lung insert, where less data was available for interpolation, lower order polynomials were applied (*e.g.* order 3 for 4 datapoints, order 4 for 5 datapoints, etc. up to order 9. Then order 9 was applied for 10 to 15 datapoints to avoid overfitting). This way, the expected film signal between the film layers could be determined, allowing for the reconstruction of the 3D dose distribution within the lung volume at a high resolution. An example of one such dose distribution reconstructed using this algorithm is provided in Fig. 2.

#### Dosimetric verification

For each treatment delivery, the DICOM-RT dose structure was exported from the TPS and read into MATLAB. The alignment of the calculated and measured dose distributions was achieved by fixing four 0.2 mm thick circular copper markers in the corners of the central film layer during acquisition of the planning CT scans, such that the coordinates of these markers were easily identifiable in the CT scan. This central film layer was then used as a template to permanently mark the central layer of each set of films to be used for treatment delivery, allowing these same four points to be identified in the reconstructed delivery dose distribution. The two dose distributions were then spatially aligned based on the location of these markers. Once aligned based on the location of a single reference marker, the other three reference markers were usually in agreement to within 1 or 2 pixels, with the maximal alignment error observed in the horizontal and vertical directions not exceeding 3 pixels (1.016 mm at the scanned resolution of 75 dpi). As a result, alignment of the two dose distributions was based on the average relative location of these four markers.

A MATLAB function was written for 3D gamma comparison between the two matched dose distributions, with the treatment planning dose matrix considered as reference [16]. This algorithm was applied for each treatment for the gamma criteria of 3%/3 mm, 3%/2 mm, 2%/2 mm and 1%/1 mm and a dose threshold of 10%. Above this threshold, the global gamma passing rate and mean gamma value were determined and 3D spatial maps of all the gamma values associated with each treatment delivery were produced.

### Robustness of Monte-Carlo dose calculation

Due to the uncertainty associated with the Monte-Carlo dose calculation the treatment planning dose was calculated a total of three times for the 1-fiducial Synchrony-based treatment delivery with the phantom setup which did not allow for target rotation. The 3D delivered dose distribution was then compared against each of the recalculated treatment planning dose distributions for the gamma criteria of 3%/2 mm with a 10% threshold to assess the effect of the Monte-Carlo dose calculation on the resulting dose agreement.

## Results

### Dosimetric verification

**Fig. 3:**
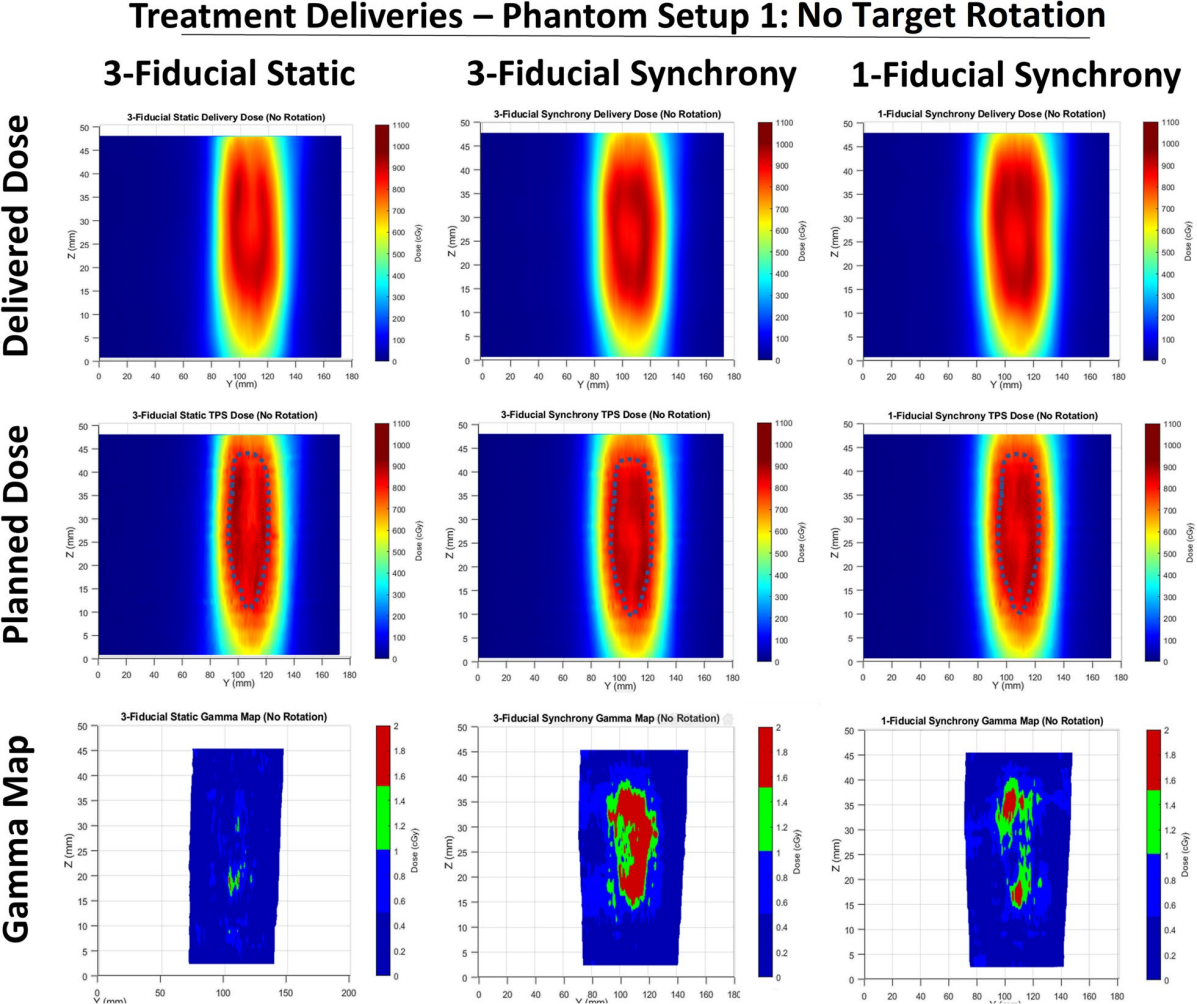
Sagittal views of the delivered and TPS-calculated dose distributions and the gamma maps for tested plans using the phantom setup with no target rotation. The Gamma criteria of (3%, 2 mm) is used

**Fig. 4:**
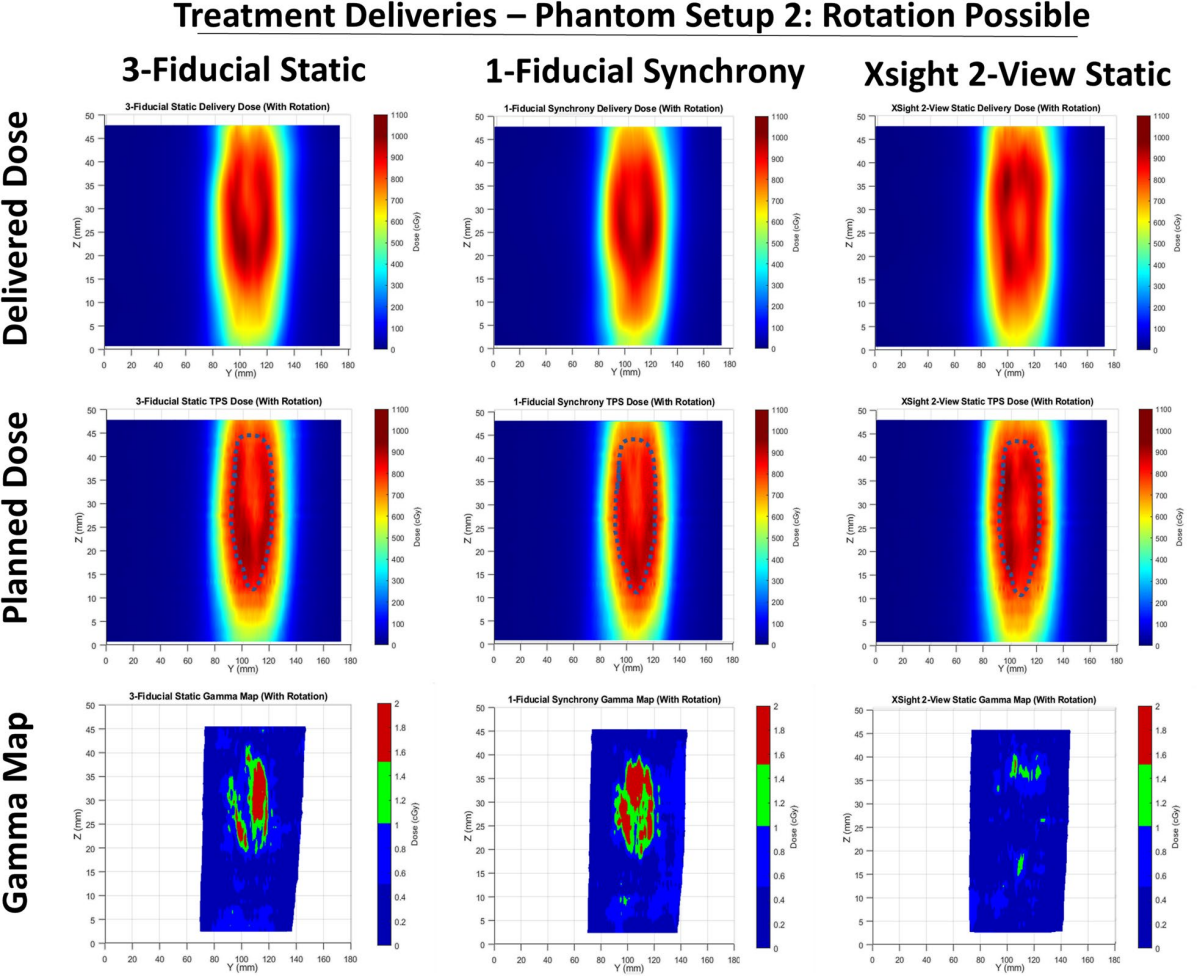
Sagittal views of the delivered and TPS-calculated dose distributions and the gamma maps for tested plans using the phantom setup with target rotation. The Gamma criteria of (3%, 2 mm) is used

**Fig. 5:**
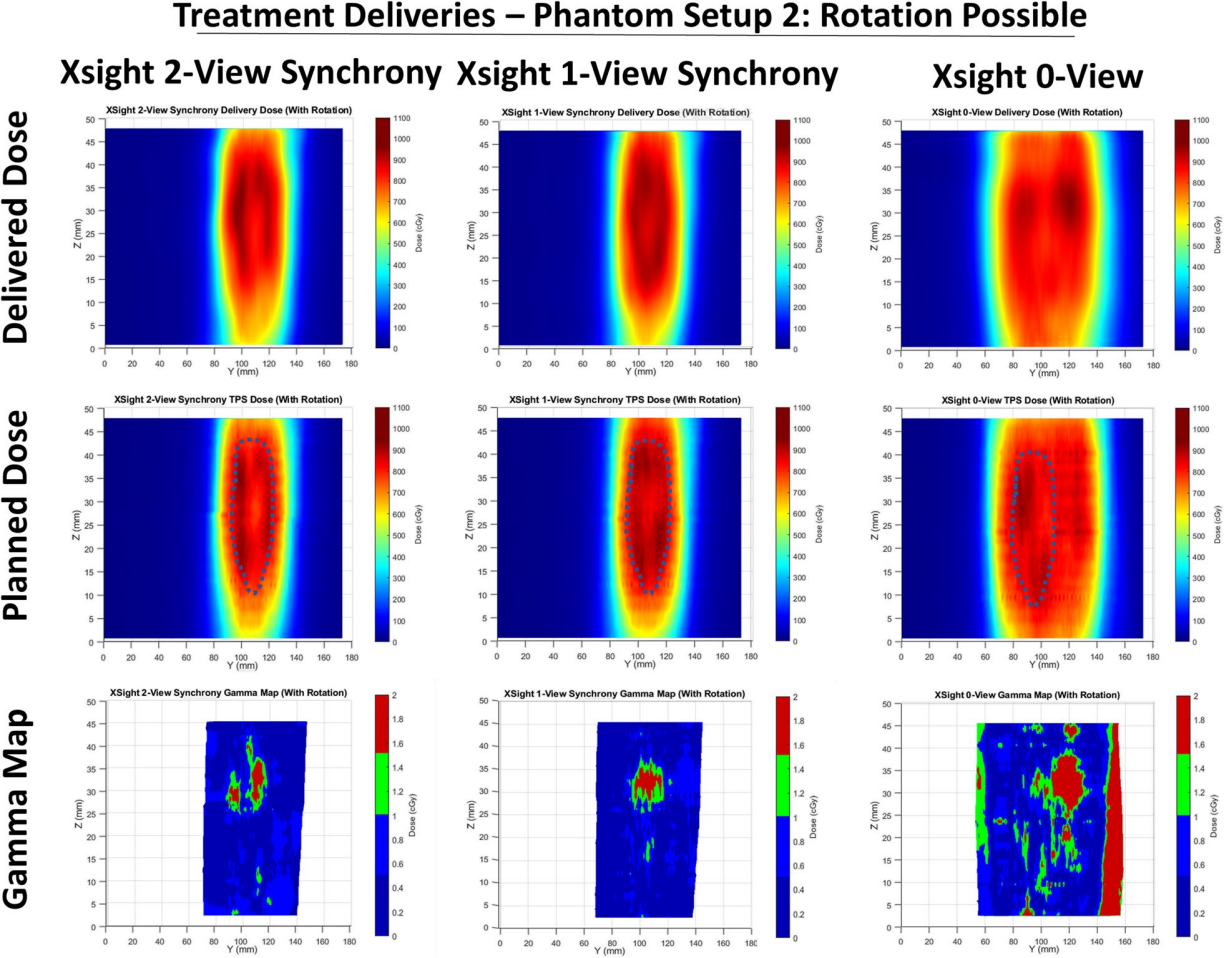
Sagittal views of the delivered and TPS-calculated dose distributions and the gamma maps for tested plans using the phantom setup with target rotation. The Gamma criteria of (3%, 2 mm) is used

Table 2 shows the gamma comparison results for all tested plans and gamma criteria. In addition, Figs. 3, 4 and 5 show the sagittal view for each of the reconstructed delivery dose distributions, the matched TPS dose distributions, and the associated gamma maps for the 3%/2 mm criteria. The location of GTV is indicated on each of the TPS dose distributions with a dotted line.

**Table 2:** Gamma pass rates and mean Gamma values for the (3%/3 mm), (3%/2 mm) and (2%/2 mm) criteria for each tested plan

Treatment modality	Gamma criteria	Gamma passing rate	Mean gamma value
3-Fiducial static (No target rotation) (FTTS)	3%/3 mm	99.8%	0.22
	3%/2 mm	99.3%	0.29
	2%/2 mm	98.6%	0.33
3-Fiducial synchrony (No target rotation) (FTTS)	3%/3 mm	96.7%	0.30
	3%/2 mm	93.7%	0.39
	2%/2 mm	92.9%	0.46
1-Fiducial synchrony (No target rotation) (FTTS)	3%/3 mm	99.2%	0.23
	3%/2 mm	97.8%	0.30
	2%/2 mm	96.9%	0.34
3-Fiducial static (Target rotation allowed) (FTTS)	3%/3 mm	98.6%	0.25
	3%/2 mm	97.1%	0.33
	2%/2 mm	96.3%	0.38
1-Fiducial synchrony (Target rotation allowed) (FTTS)	3%/3 mm	96.3%	0.36
	3%/2 mm	91.2%	0.48
	2%/2 mm	87.8%	0.57
XLTS 2-view static (Target rotation allowed)	3%/3 mm	99.9%	0.22
	3%/2 mm	99.4%	0.28
	2%/2 mm	98.4%	0.33
XLTS 2-view synchrony (Target Rotation Allowed)	3%/3 mm	97.3%	0.35
	3%/2 mm	92.8%	0.46
	2%/2 mm	89.2%	0.54
XLTS 1-view synchrony (Target ROTATION ALLOWED)	3%/3 mm	98.5%	0.29
	3%/2 mm	96.2%	0.38
	2%/2 mm	93.44%	0.44
XLTS 0-View (Target rotation allowed)	3%/3 mm	85.0%	0.55
	3%/2 mm	76.1%	0.70
	2%/2 mm	69.7%	0.88

### Robustness of Monte-Carlo dose calculation

The gamma pass rates observed when the delivered dose distributions were compared against each of the repeated TPS dose distributions were found to be 97.82%, 97.56% and 97.66% for the 3%/2 mm gamma criteria. The average gamma pass rate of all three dose calculations was found to be 97.68% with a standard deviation of 0.13%.

## Discussion

This study presents the development and evaluation of a novel dosimetric method for assessment of the accuracy of the XLTS tumour tracking system on the CyberKnife machine by comparison to the FTTS system for various target motions and modalities.

### Number of fiducial markers

The data presented in Table 2 showed that when the target was not allowed to rotate, the gamma pass rate for the single fiducial Synchrony-based treatment delivery was 97.82%, comparable to results observed for the static deliveries and exceeding the performance of the 3-fiducial Synchrony-based treatment delivery by 4.08% for the 3%/2 mm gamma criteria. This was expected since the target motion was restricted to superior-inferior direction and therefore, the benefit of applying rotational corrections using 3-fiducial markers was irrelevant. In particular, during the FTTS treatment delivery with 3 fiducial markers, the system was unable to exactly match onto the fiducial array in a number of kV images, predominantly for the two most inferior markers which were 16 mm apart. In such cases, the system reported a correlation model error in the range of 3–6 mm, resulting in erroneous data points being added to the Synchrony model. In this study, the operator had to manually remove or rebuild the Synchrony model in the case where the 5 mm correlation model error tolerance was exceeded, which was the PTV margin used in this study. In addition, in these cases, the system would often recommend the application of unnecessary translational and rotational corrections to better match onto the fiducial locations for treatment delivery, however, upon applying such corrections the fiducial matching often worsened. The problem was attributed to the limitation of the phantom used for this investigation. The phantom dimensions with added spine and ribs made it impossible to pick the fiducials out in parts of the breathing cycle with rotation, despite the imaging settings having been optimised as much as possible. Thus, it was chosen to monitor and adjust model to limit the correlation error to within the PTV margin used.

In the case of 1-fiducial tracking for the same phantom setup, the FTTS found it easier to lock onto the top-most fiducial for target-tracking without any of the aforementioned problems and consequently, this was likely a contributing factor to the better dose agreement observed for the 1-fiducial Synchrony-based delivery when compared to the 3-fiducial Synchrony-based delivery with the same setup. Such errors could potentially have arisen due to differences in the relative positions of the markers within the fiducial array during delivery when compared to the treatment plan. In this study, however, fiducial drifting was not possible, and the phantom alignment was carefully carried out according to clinical protocols prior to treatment delivery.

When the target was allowed to rotate, the dose agreement for the 3%/2 mm gamma criteria when tracking with only a single fiducial marker was degraded from 97.82% to 91.24%. This result is expected as when tracking with only a single fiducial marker the system is unable to account for the rotational motion of the target during delivery. Notably, however, the 3-fiducial Synchrony treatment delivery for this phantom setup could not be completed. This occurred as the rotational corrections required by the Synchrony system to accurately match onto the 3-fiducial array were observed to exceed the tolerances set out by the Synchrony system at various points throughout the respiratory cycle, preventing the treatment from being completed. This result has some notable connotations, as the 1-fiducal treatment delivery was able to proceed unhindered, with the reported correlation model error never approaching a level which would raise any concerns to the operator, suggesting that by using 3 fiducials to facilitate target tracking, delivery errors which might go unnoticed with 1-fiducial tracking could be mitigated. This explains the degradation observed in the gamma comparison result for the 1-fiducial Synchrony treatment delivery, as despite the significant degree of target rotation, the delivery was not interrupted, resulting in deviations between the delivered radiation dose when compared to the TPS prediction.

It was noted that with larger target rotations, the dose delivery accuracy afforded by tracking with 3 fiducial markers was likely to exceed what was achievable using a single fiducial marker for target tracking. Despite this, in the absence of target rotation, for the target and motion considered in this study, a comparable accuracy was observed for the 1 and 3 fiducial tracking modalities. Consequently, based on results of this work it is recommended that for minimal target rotations where the advantages of tracking with 3 fiducial markers are insignificant, the option of employing tracking with a single gold seed be considered, as the benefits associated with 3-fiducial tracking may not outweigh the detriment associated with the insertion of the additional 2 fiducial markers in such cases. To confirm this hypothesis, further deliveries covering a range of target geometries, motions and rotations would need to be considered, as in this study only a single patient model was observed. In selected patients at a particularly high risk of pneumothorax, tumour rotation can be analysed prior to insertion of a fiducial marker with a 4D respiration correlated CT scan, which would allow an assessment of tumour rotation, followed by an informed decision on the most suitable target tracking modality based on the degree of target rotation. However, other advantages of 3-fiducial tracking must also be considered, including the ability to tell if one fiducial marker has migrated relative to the other fiducials in the planning CT scan.

Other authors, such as Subedi et al. [17] have recommended against treating patients with a single fiducial marker. Notably, the authors made this recommendation on the basis that when fewer fiducials were used for target-tracking, the system was found to report a higher confidence when falsely locking onto image noise while the use of greater number of fiducials led to increased precision of targeting for true locks. But to induce false locks, the authors had intentionally degraded the image quality so that they could assess the effect of image quality on targeting accuracy [17]. However, in the present study clinical protocols were followed such that the image quality was optimized for visualisation of the fiducial markers. Therefore, false locks onto image noise were not expected to have a significant effect on the targeting accuracy and were not observed when target-tracking was employed with a single fiducial marker.

### XLTS 2-view vs. 1-view tracking

All of the tested XLTS-based deliveries in this study were performed using the phantom setup which allowed for target rotation. From the results presented in Table 2, the XLTS 1-view Synchrony treatment delivery showed better dose agreement than the XLTS 2-view Synchrony by 3.44% for the gamma criteria of 3%/2 mm, which is a surprising result, as one would expect higher accuracy in target localisation and consequently higher dose delivery accuracy when the target could be adequately visualised and tracked by both of the X-ray imagers. Despite the XLTS 1-view demonstrating comparable accuracy to the XLTS 2-view, the latter would be preferable, as in clinical practice a smaller PTV expansion margin is used for the XLTS 2-view compared to the XLTS 1-view. The result may be due to differences in the targeting accuracy between the two X-ray imagers, which may have occurred due to the different imaging parameters which were optimised for each of the X-ray images during treatment simulation. During treatment simulation, 8 images were acquired in each of the X-ray imagers to ensure adequate visualisation of the tumour and while the tumour was adequately visualised in all 8 images with one camera, the target was accurately localised in only 7/8 (87.5%) of the images acquired with the second Synchrony camera. However, as only 1 image failed the simulation, the 2-view treatment delivery was able to proceed. Consequently, during delivery as an XLTS 2-view, it is possible that for certain repeatable positions in the respiratory cycle, the target may not have been accurately localised by the X-ray imager which exhibited poorer simulation results, leading to the addition of erroneous points into the Synchrony model, which may go unnoticed by the operator, degrading the accuracy of dose delivery. In particular, when compared to the FTTS, the XLTS was less accurate in identifying the target position when the most inferior half of the target was obscured by the overlying bony rib structure, which occurred predominantly in only one of the X-ray imagers. Nakayama et al. calculated the correlation and prediction model uncertainties associated with the XLTS 2-view and 1-view treatment deliveries [12]. They found no differences in error between the XLTS 2-view and 1-view tracking modalities; however, it should be noted that such analysis performed through the CyberKnife log-files cannot provide an independent measure of the dose-delivery accuracy, since log-files are generated by the system itself [12].

### Accuracy of tracking with XLTS compared with FTTS

With the exception of the XLTS 0-View treatment delivery, this study showed that the accuracy of the XLTS was comparable to, and even higher than FTTS for treatments planned and delivered using the same phantom setup. As all XLTS deliveries were performed using the phantom with target rotation, the XLTS has demonstrated the ability to deliver the desired treatment planning dose distribution with a high degree of geometrical accuracy for an irregular patient breathing pattern in the presence of target rotation under realistic treatment conditions. This result is in agreement with findings by Jung et al. [14], who reported that the segmentation accuracy of the XLTS was comparable to that of the FTTS through analysis of the log-files generated by the CyberKnife system during treatments delivered to a lung phantom utilizing 1 fiducial marker for target tracking in addition to the XLTS 2-view target tracking modality [14]. Jung et al. also performed measurements of the 2-D dose distributions through the central plane of the lung for each of these treatment deliveries. The dose distributions measured for fiducial-based target-tracking utilizing a single gold seed were compared against the corresponding XLTS 2-view treatment delivery and the average gamma pass rate for the 3%/3 mm, 2%/2 mm and 1%/1 mm criteria were found to be 100%, 99.6% and 86.8%, respectively [14]. Despite the apparent excellent results observed in this study, it should be noted that Jung et al. [14] conducted target-tracking utilizing only a single gold seed with a phantom setup which did not include the spine. Therefore, spine alignment would not have been possible, and the authors would have been unable to apply rotational corrections prior to or throughout the course of treatment delivery. As a result, it would have been necessary for the authors to exactly recreate the phantom setup for treatment delivery without the aid of a spine setup for phantom alignment and a number of assumptions would have to be made about the phantom setup and alignment, which would not be possible in a clinical environment. In their study, treatments were delivered using an isocentric beam delivery technique with a fixed 30 mm collimator, which is an oversimplified beam geometry and not clinically used, as it would not be possible to achieve sufficient target coverage except for the case where the target was perfectly spherical (or close to it).

In the present study, all margins were defined to keep with the current clinical practice of stereotactic lung protocol. A 5 mm margin is standard for single fiducial where fiducial is within the PTV and XLTS, as it represents the maximum allowable Synchrony model error for an individual point. A 7 mm margin was used for 1-view lung in untracked direction is to allow for unexpected untracked motions. The margins are based on the assumption that the tumour tracking performance is ideal, but results of this study indicate that it may not always be the case.

### Dosimetric comparison

The sagittal dose distributions in Figs. 3, 4 and 5 showed that for all Synchrony-based deliveries, higher doses were delivered in the high-dose region, in some cases in excess of 100 cGy higher than the treatment plan, which was a contributing factor to the dose delivery error for all treatment deliveries. Considering that the uncertainty in film dosimetry process was evaluated and was only 0.6%, to ensure that the reason was not the film scanner system, measurements were repeated and an alternate film scanner was used which provided similar results. The other potential cause would be the QUASAR respiratory motion phantom which has a thickness of only 120 mm, and consequently, as lung insert moves superiorly and inferiorly it is possible that the most superior and inferior beams which traverse through the phantom in the treatment plan may instead pass through regions of air above and below the insert during treatment delivery. This would result in decreased attenuation and consequently higher dose delivered to the PTV. This effect would have been present for both XLTS and FTTS based treatment deliveries but would not affect real patients.

### Effect of Monte-Carlo dose calculation uncertainty

The average gamma pass rate of all three dose calculations was found to be 97.68% with a standard deviation of only 0.13%. Therefore, the impact of the variability in the Monte-Carlo dose calculation algorithm on the resulting gamma pass rates was small and unlikely to have any significant effect on the results.

## Conclusion

A modified phantom and 3D film dosimetry method were developed to compare the accuracy of dose delivery in CyberKnife treatments involving fiducial-based and soft-tissue tumour tracking systems. This method was used to compare the entire delivered 3D dose distributions with the TPS-calculated dose distributions within the lung volume for NSCLC treatments using the CyberKnife FTTS and XLTS under clinical treatment conditions. This study confirmed that the dose delivery accuracy of the XLTS is comparable to, and even higher than, that of the FTTS, highlighting the feasibility of fiducial-free target tracking for NSCLC patients where the target can be adequately visualised. In some patients, with minimal target rotation, a single fiducial marker may be more suitable than three due to the detriment associated with the fiducial insertion procedure.

## Data Availability

No data are available for sharing.

## Abbreviations

SABR: Stereotactic ablative radiation therapy
NSCLC: Non-small-cell lung-cancer
XLTS: Xsight lung tracking system
FTTS: Fiducial-based target-tracking systems
